# Impact of Epstein Barr Virus Infection on Treatment Opportunities in Patients with Nasopharyngeal Cancer

**DOI:** 10.3390/cancers15051626

**Published:** 2023-03-06

**Authors:** Francesco Perri, Francesco Sabbatino, Alessandro Ottaiano, Roberta Fusco, Michele Caraglia, Marco Cascella, Francesco Longo, Rosalia Anna Rega, Giovanni Salzano, Monica Pontone, Maria Luisa Marciano, Arianna Piccirillo, Massimo Montano, Morena Fasano, Fortunato Ciardiello, Giuseppina Della Vittoria Scarpati, Franco Ionna

**Affiliations:** 1 Medical and Experimental Head and Neck Oncology Unit, INT IRCCS Foundation G. Pascale, 80131 Napoli, Italy; 2 Medical Oncology Department, University of Salerno, 84084 Fisciano, Italy; 3 SSD Innovative Therapies for Abdominal Metastases, Department of Abdominal Oncology, INT IRCCS Foundation G. Pascale, 80131 Napoli, Italy; 4 Medical Oncology Division, IGEA SPA, 41012 Naples, Italy; 5 Department of Precision Medicine, University of Campania Luigi Vanvitelli, 81100 Caserta, Italy; 6 Division of Anesthesia and Pain Medicine, INT IRCCS Foundation G Pascale, 80131 Napoli, Italy; 7 Otolaryngology and Maxillofacial Surgery Surgery Unit, INT IRCCS Foundation G Pascale, 80131 Napoli, Italy; 8 Maxillofacial Surgery Surgery Unit, Reproductive and Odontostomatological Science, University of Naples Federico II, 80138 Napoli, Italy; 9 Medical Oncology Unit, Hospital Sir Apicella ASL NA3, Pollena Trocchia (Naples), 80040 Naples, Italy

**Keywords:** Epstein Barr Virus, nasopharyngeal carcinoma, tumor associated antigens, immunotherapy, tumor microenvironment, check-point inhibitors

## Abstract

**Simple Summary:**

Epstein Barr virus (EBV) is often responsible for the onset of both solid and hematologic malignancies. In particular, it is implicated in the pathogenesis of nasopharyngeal carcinomas (NPCs). Some viral proteins produced during the latent phase of the virus itself in epithelial cells induce or promote carcinogenesis. These oncoproteins can be used as targets of various immunotherapy strategies. In the past, active or adoptive immunotherapy techniques have been employed. Recently however, the use of check-point inhibitors seems very promising. Some check-point inhibitors are already in use in clinical practice, others will soon receive regulatory approval.

**Abstract:**

Chemical, physical, and infectious agents may induce carcinogenesis, and in the latter case, viruses are involved in most cases. The occurrence of virus-induced carcinogenesis is a complex process caused by an interaction across multiple genes, mainly depending by the type of the virus. Molecular mechanisms at the basis of viral carcinogenesis, mainly suggest the involvement of a dysregulation of the cell cycle. Among the virus-inducing carcinogenesis, Epstein Barr Virus (EBV) plays a major role in the development of both hematological and oncological malignancies and importantly, several lines of evidence demonstrated that nasopharyngeal carcinoma (NPC) is consistently associated with EBV infection. Cancerogenesis in NPC may be induced by the activation of different EBV “oncoproteins” which are produced during the so called “latency phase” of EBV in the host cells. Moreover, EBV presence in NPC does affect the tumor microenvironment (TME) leading to a strongly immunosuppressed status. Translational implications of the above-mentioned statements are that EBV-infected NPC cells can express proteins potentially recognized by immune cells in order to elicit a host immune response (tumor associated antigens). Three immunotherapeutic approaches have been implemented for the treatment of NPC including active, adoptive immunotherapy, and modulation of immune regulatory molecules by use of the so-called checkpoint inhibitors. In this review, we will highlight the role of EBV infection in NPC development and analyze its possible implications on therapy strategies.

## 1. Background

Carcinogenesis is a multistep process that consists of the transformation of healthy cells into cancer cells. This process goes through three different phases: initiation, promotion, and progression. In the first stage, a genetic mutation predisposes to the development of cancer. During the second stage, the accumulation of other genetic mutations allows the cells to acquire a cancerous phenotype. Finally, in the progression phase, the further accumulation of mutations results in genetic heterogeneity as well as the acquisition of a more aggressive phenotype and the development of cellular polyclonality [1]. Chemical, physical, and infectious agents may induce carcinogenesis. Viruses are the major causes of infectious-induced carcinogenesis [2]. The occurrence of *virus*-induced *carcinogenesis* is a complex process caused by an interaction across multiple genes, mainly depending by the type of the virus. Mechanisms underlying virus-induced *carcinogenesis* are not clearly established. Molecular mechanisms mainly suggest the involvement of a dysregulation of the cell cycle [3]. Among the virus-inducing *carcinogenesis,* Epstein Barr Virus (EBV) plays a major role in the development of both hematological and epithelial malignancies [4,5,6]. EBV belongs to the gamma-herpesviridiae family and it is the causative agent of infectious mononucleosis. Several lines of evidence demonstrated that nasopharyngeal carcinoma (NPC) is consistently associated with *EBV* infection. Specifically, about 95% of nonkeratinizing and undifferentiated NPCs are associated with EBV although, like other solid tumors, genetic susceptibility and environmental factors also contribute to their development. EBV is found in both malignant and premalignant lesions of nasopharynx [7]. In addition, numerous studies reported an elevated anti-EBV antibodies titers and the presence of EBV DNA in nearly all endemic undifferentiated variant of NPC [8,9].

Although EBV efficiently infects and induces growth transformation of epithelial cells in vivo, its ability are much less efficient in vitro [10]. To enter host cells, EBV uses a glycoprotein named Gp350/20, which in turn interacts with the complement receptor 2 (CR2/CD21). The latter is a membrane-associated protein that plays a key role in B lymphocyte function. Nevertheless, CD21 is also expressed by epithelial cells of both nasopharynx and oropharynx [8]. Once infected host cells, EBV rapidly replicates and gets out of the infected cells causing their lysis (lytic phase). Nevertheless, EBV can also enter a dormant phase, named “latency”. During this phase, characterized by a partial expression of the viral antigens, three forms of latency (namely “type I, type II, and type III” latency) [11] can be distinguished based on the number of the expressed antigens.

Neoplastic transformation induced by EBV is most likely caused by the latent phase rather than the lytic phase. During the latent phase, EBV is not silent and multiple viral genes are activated. Some of these genes can promote cancerogenesis in presence of host cell DNA mutations. Products of these viral genes are proteins as well as macromolecules, which play an important role in the permanence of EBV in host cells as well as in the transmission of viral DNA into daughter cells during mitosis [12]. As a result, these macromolecules and especially those with a protein structure are considered as viral oncoproteins and, even more, as tumor-associated antigens (TAAs) in the host infected cells. TAAs are major players for eliciting a host immune response.

In this review, we will highlight the role of EBV infection in NPC development and analyze its possible implications on therapy strategies.

## 2. EBV-Induced Carcinogenesis in NPC

**Figure 1:**
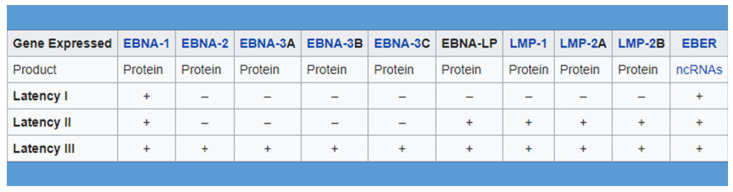
There are three forms of latency expressed by EBV-infected cells, each one characterized by a unique expression of EBV-associated proteins and RNAs.

During EBV latent infection phase, expression of the full viral genome is restricted and few viral genes are expressed, leading the virus to escape to its recognition and destruction by the host immune system as well as to remain silent within the host cell. As we mentioned before, there are three forms of latency expressed by EBV-infected cells, each one characterized by a unique expression of EBV-associated proteins and RNAs. Type I latency is associated with the expression of one viral antigen, namely EBNA1 (EBV nuclear antigen-1); type II latency by the expression of EBNA1, LMP1/2 (latent membrane protein) and EBERs (EBV–encoded small RNAs). Lastly, type III latency is associated with the greatest number of viral antigens, namely EBNA 1, 2, and 3, EBNA-LP, LMP-1 and 2, and EBERs (Figure 1) [13,14,15,16]. Type II latency is acknowledged to be associated with Hodgkin’s lymphoma and NPC [15,16].

According to some lines of evidence [17,18], following a first phase of EBV infection of peripheral blood B-lymphocytes, EBV reactivates in B-lymphocytes and especially in those present in the tonsillar Waldeyer’s ring. Infected B -lymphocytes release several virions. As a result, virus directly sheds from lymphocytes into epithelial cells of nasopharynx, establishing a latent infection, which can lead to cancerogenesis.

Cancerogenesis may be induced by the activation of different EBV “oncoproteins” such as EBNA1, LMP1, and LMP2. EBNA1 binds to the origin of replication of the viral genome and enables the viral episome to segregate with the host chromosomes during mitosis. EBNA1 can also lower the nuclear levels of P53, leading to an impaired DNA repair and genetic instability [19,20,21]. P53, better known as “the guardian of genome” plays a crucial role in regulating cell cycle and promoting DNA-damage repair. In case of DNA-damage, P53 blocks the cell cycle and stimulates DNA-damage repair and when the damage cannot be repaired, P53 stimulates apoptosis, thus preventing DNA damage from being perpetrated on daughter cells. When P53 is down regulated, DNA-mutations could be acquired by cells inducing carcinogenesis [22]. LMP-1, like other oncogenes, represents a key transforming protein. It inhibits the differentiation of epithelial cells, stimulating their growth by promotion of cell cycle progression. Furthermore, LMP-1 also stimulates the expression of the epidermal growth factor receptor (EGFR), promoting cell growth through activation of the MAP-Kinase pathway [23,24,25]. In addition, in epithelial cells, LMP1 inhibits P53-mediated induction of apoptosis, and more importantly, induces the sensibilization of lymphocytes to TGF-beta, dampening the immune response against cancer cells [26]. Lastly, two isoforms of LMP2 exist, namely LMP2A and 2B. Both are able to initiate oncogenic transformation in EBV-infected cells through the activation of protumorigenic signaling pathways such as promotion of cancer cell division and migration by activation of the Ras/PI3K/Akt and the β-catenin/Wnt pathways by LMP2A [27,28]. In summary, it can be stated that EBV viral oncoproteins have the ability to induce carcinogenesis although they need further alterations in both oncogenes and tumor suppressor genes of infected cells.

## 3. Impact of EBV-Infection on the Tumor-Microenvironment of NPC

**Figure 2:**
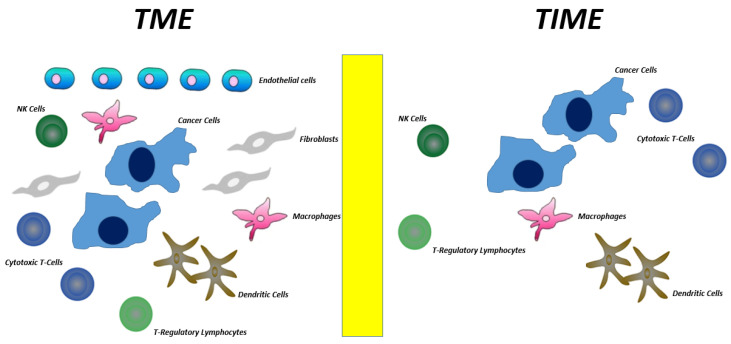
Differences between TME and TIME.

The tumor microenvironment (TME) is the region surrounding the tumor cells. It includes stromal cells such as fibroblasts, endothelial cells, and immune cells as well as non-cellular components such as the extracellular matrix. On the other hand, the tumor immune microenvironment (TIME) refers only to the immune component of the TME and does not include the stromal and extracellular components [29,30] (Figure 2). Both immunostimulating and immunosuppressive cells are present in TIME; the former stimulates an antitumor immune response, the latter inhibits the antitumor immune response and promotes tumor progression. Composition of the TIME strongly depends on the cytokines production during cancer progression. First, cancer cells stimulate an innate and aspecific response mediated by IL-1, IL-6, and TNF-alpha production; this kind of response mainly stimulates macrophages and neutrophil granulocytes to attack the tumor cells. Nevertheless, this aspecific response is not efficient enough at fighting cancer cells. Therefore, then, mainly due to dendritic cells engulfing and exposing TAAs, an adaptive immune response characterized by cytokines production from helper T-lymphocytes is induced. If “the game is conducted” by the TH1 helper lymphocytes (inflammatory), a robust anti-cancer immune response develops and cytokines such as IL-12, IL-2, and IFN-gamma mainly stimulate cytotoxic T-lymphocytes (CTLs) and natural killer cells (NKs) [31,32]. On the other hand, if the mix of cytokines produced (mainly by cancer cells) stimulates the generation of T-helpers other than TH1, for example TH2, Th17, and/or T-Regs (regulatory), the result will be a particularly immunosuppressed TIME, namely rich in T-Regs, M1 macrophages, and myeloid-derived suppressor cells (MDSCs).

Overall, in squamous cell carcinoma of the head and neck (SCCHN), TIME mainly contains immunosuppressive cells such as T-regulatory lymphocytes (T-Regs), M1 macrophages, and myeloid-derived suppressor cells (MDSCs). In contrast, a very low number of immunostimulanting cells such as TH1 helper lymphocytes, CTLs, and NKs are present. In NPC, TIME also exhibits mainly an immunosuppressive infiltration and, even more, EBV-positive NPC displays a higher immunosuppressive TIME as compared to that of EBV negative NPC [30,33]. In NPC, besides the above-mentioned immunosuppressive cells, TIME also contains two unique cell subtypes, namely LAMP3 dendritic cells and M1-M2 macrophages. LAMP3 dendritic cells express on their membrane both cytotoxic T-lymphocyte-associated protein 4 (CTLA-4) and programmed death 1 ligand (PD-L1). Their interaction by CTLA-4 on Tregs and PD-L1 by CTLs induces down-regulation of antigen processing/presentation and induction of energy, respectively [30,34,35]. M1-M2 macrophages are cells displaying an intermediate phenotype between tumor-suppressing M1 and tumor-promoting M2 subtypes. M1-M2 macrophages produce a high quantity of inflammatory cytokines such as IL-1, IL-6, and TNF-alpha that in turn stimulate the further recruitment of other macrophages and concomitantly increase the aspecific response in site of the adaptive anti-cancer immune response. From a molecular viewpoint, production of these cytokines is mediate by the constitutive activation of NF-κB signaling (prompted by LMP1 oncoprotein) [31]. The final result is a stimulation of chronic and non-specific inflammation, mainly characterized by the prevalence of macrophages and neutrophil granulocytes instead of dendritic cells. This results in a reduced presentation of TAAs by dendritic cells to naïve T lymphocytes and ultimately in a lower production of CTLs and NKs.

## 4. Translational Implications

EBV-infected NPC cells can express proteins potentially recognized by immune cells in order to elicit a host immune response. In addition, NPC cells express numerous immune regulatory molecules such as PD-L1, CD40, CD70, CD80, and CD86 which modulate T-cell activity [36,37,38,39]. Lastly, viral antigen expression by EBV infection can also induce the expression of TAAs. On this basis, three immunotherapeutic approaches have been implemented for the treatment of NPC including active and adoptive immunotherapy and modulation of immune regulatory molecules.

Active immunotherapy consists in delivering specific TAAs in the form of therapeutic vaccines with the aim to enhance their recognition on cancer cells by the immune system and ultimately the elimination of malignant cells. Adoptive immunotherapy aims to directly activate effector cells such as CTLs and NKs [40,41].

### 4.1. Active Immunotherapy

It consists in administering therapeutic vaccines to patients affected by NPC. Two strategies can be adopted including administration of EBV-associated antigens and administration of adequately “loaded” or “stimulated” dendritic cells (DCs) with associated EBV antigens. DCs are the most efficient antigen presenting cells (APCs) able to present EBV antigens to CTLs. In different pre-clinical reports, authors have shown that DCs are able to expand a functional population of CD8+ T-cells specific to EBV-antigens [42,43,44]. One of the first therapeutic anti-EBV vaccine has employed EBV-pulsed DCs. Lin et al. treated 16 patients with recurrent chemo-refractory NPC with four injections at weekly intervals of a vaccine consisting of autologous DCs. Autologous monocyte-derived dendritic cells were cultured from patients with advanced NPC, matured with cytokine, pulsed with HLA-A1101-, A2402-, and B40011-restricted epitope peptides from EBV-LMP2 and subsequently injected into inguinal lymph nodes. Specific CTL responses were elicited or boosted in 9 patients receiving the vaccine, achieving partial tumor reduction in 2 patients [43]. Based on these results, further trials have been implemented with the aim to improve the entity of the elicited specific T-cell mediated response. One approach utilized the transfection of autologous DCs with viral vectors containing EBV antigens. The results demonstrated an increased targeted T-cell proliferation of both CD4+ and CD8+ T-cells, but no significant improvement of clinical responses [45,46,47]. In an alternative approach, virus encoding EBV-antigens were directly administered. This modified vaccinia virus Ankara (MVA) expressing a fusion gene of the CD4+ T-cell epitope-rich domain of EBNA1 and a full-length LMP2 was administered to 18 patients with advanced NPC, in the contest of a phase I trial. The drug was well tolerated and it induced differentiation and functional diversification of responsive T-cells specific for EBNA1 and LMP2 [48]. Phase II clinical trials assessing the rate of clinical responses of this type of vaccine are ongoing. 

Further vaccines were also made using EBV antigens (EBNA1, LMP2, and LMP1) [49,50] fused together or with adjuvant proteins (heat shock proteins) [51], having the purpose of eliciting a specific TH1 and CTLs response. However, no data about the obtained clinical responses have been reported.

### 4.2. Adoptive Immunotherapy

This strategy consists in “ex vivo” stimulation of autologous CD8+ T cells followed by intravenous reinfusion of the obtained mature-CTLs into the patient. Straathof et al. carried out a phase I dose escalation clinical trial including ten patients with advanced-chemorefractory NPC treated with autologous EBV-restricted CD8+ lymphocytes. Patient peripheral blood mononuclear cells (PBMCs) were stimulated with autologous irradiated EBV lymphoblastoid cells (LCLs). This process aimed to enhance the expression of EBV antigens. PBMCs were then co-cultured with EBV-specific CTLs to allow their maturation. Lastly, CTLs were reinfused in the patients. The results of the trial demonstrated that the injection was well-tolerated. As a result, a phase II trial was implemented; adding further 13 patients to patients already included in the phase I trial [52,53]. An overall survival (OS) of 87% and 70% at 1- and 2-years, respectively, as well as a good response rate was observed.

Generation of CTLs from peripheral blood is difficult, expensive, and time consuming. In fact, the first step consists in the maturation of PBMCs into mature CTLs and this requires their stimulation with LCLs and interleukin-2; to obtain the latter, further expensive procedures are required. Maturation of CTLs may not occur and if it does, it takes time. As a result, in order to implement an effective adoptive immunotherapy some authors also tried to isolate CTLs from the TME, utilizing tumor-infiltrating lymphocytes (TILs). Li et al. [54] reported the results of a trial where 20 patients with locally advanced NPC were treated with a combination of chemotherapy and adoptive TIL-based immunotherapy. In particular, 20 patients received a single-dose of TIL infusion following concomitant chemoradiation (cisplatin plus curative-intent radiation therapy). As result, nineteen of 20 patients experienced an objective antitumor response (overall response rate: 95%), and 18 patients displayed disease-free survival longer than 12 months. The combination therapy showed a good response rate and a good efficacy; however a clear benefit from immunotherapy was not quantifiable since patients were also treated with chemo-radiotherapy.

### 4.3. Immune Checkpoint Inhibitors

**Figure 3:**
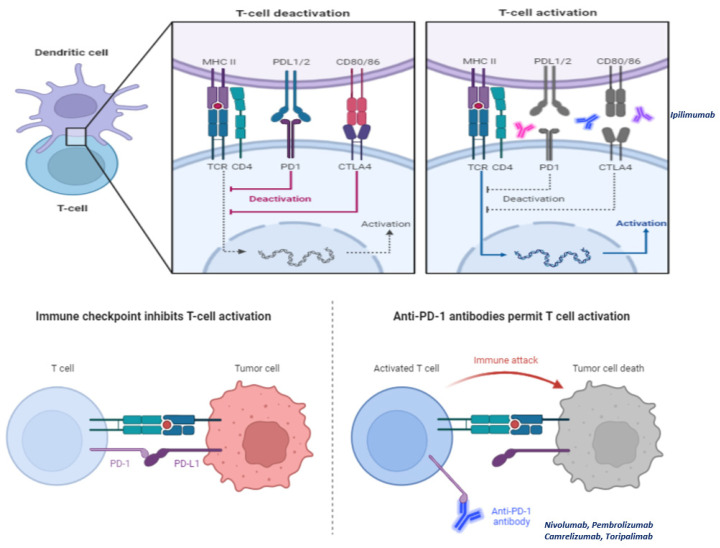
Check-point inhibitors act by removing the so-called “inhibitory brakes”, namely stimuli elicited through particular ligands (CTLA-4 and PD-1 for example) which lead cytotoxic T lymphocytes (CTLs) to anergy. The molecules best known and used in clinical practice are CTLA-4 inhibitors (ipilimumab) and PD-1 inhibitors (nivolumab, pembrolizumab, camtelizumab, and toripalimab).

TIME of NPC, especially those who are EBV related, is strongly immunosuppressed due to the special pattern of cytokines produced by both immune and tumor cells [30,33,35]. On the other hand, NPC cells express numerous immune regulatory molecules (defined as immune checkpoints) which can be targeted. On this basis, it has been hypothesized that the use of checkpoint inhibitors can achieve therapeutic benefits in patients with EBV-related NPC. As we mentioned before, both immune checkpoints CTLA-4 and PD-L1 exert an inhibitory signal on lymphocytes (especially on Treg and CTLs). Checkpoint inhibitors act by removing these inhibitory brakes, leading to stimulation of lymphocytes and in turn, tumor-cells destruction (Figure 3. NPC are characterized by high PD-L1 expression and intense infiltration of lymphocytes. This makes checkpoint inhibitors a promising treatment option [55,56]. Monoclonal antibodies targeting CTLA-4 or PD-1/PDL-1 axis is available. Data regarding anti-PD-1/PDL-1 are more consistent than those obtained by anti-CTLA-4 for NPC patients. Some of anti-PD-1 antibodies have been also already approved and are currently utilized in clinical practice for the treatment of NPC patients.

Nivolumab, a monoclonal antibody targeting the PD-1 receptor, was tested in platinum-refractory recurrent/metastatic patients with SCCHN including NPC in the context of a phase III randomized trial. Administration of nivolumab was compared to standardized second-line chemotherapy, based on the choice of the experimenter [57]. Nivolumab administration demonstrated to significantly prolong OS as compared to standard chemotherapy, leading to its approval by regulatory authorities. The indication for the use of nivolumab was subsequently also extended to the subgroup of patients with recurrent/metastatic platinum-refractory NPC.

Camrelizumab, another monoclonal antibody directed against PD-1, has shown encouraging results both in terms of activity and efficacy in phase I and II trials, especially when added to the combination of cisplatin and gemcitabine. The median objective response rate (ORR) reported was near to 90%. Median PFS rate at 12-month was about 60% [58,59]. Subsequently, Yang et al. carried out a phase III randomized trial, comparing administration of cisplatin-gemcitabine to that of the combination of camrelizumab and cisplatin-gemcitabine, in patients with platinum-sensitive recurrent/metastatic NPC. PFS, main endpoint of the study, was significantly longer in the camrelizumab group (median 9.7 months) as compared to chemotherapy alone (median 6.9 months, hazard ratio 0.54, *p* = 0.0002) [60]. Both ORR and toxicity did not significantly differ between the two treatment arms. 

Toripalimab is a humanized IgG4K monoclonal antibody targeting PD-1. Toripalimab has shown encouraging results in phase I and II trials, both alone and in combination with cisplatin-gemcitabine in platinum-sensitive patients with advanced NPC [61,62]. In an international, double-blind, randomized, placebo-controlled phase III trial, Mai et al. compared the administration of cisplatin-gemcitabine to that of the combination of toripalimab and cisplatin-gemcitabine in patients with recurrent/metastatic platinum-sensitive NPC [63]. A significant improvement in PFS was reported in the toripalimab arm compared to chemotherapy alone. Median PFS was 11.7 and 8.0 months for toripalimab plus chemotherapy and chemotherapy alone, respectively (hazard ratio  =  0.52, *p* =  0.0003). In addition, the ORR was significantly higher in the toripalimab arm (77.4%) than in the placebo arm (66.4%), (*p*  =  0.0335). A subgroup analysis, in the context of the same trial, revealed that the subgroup of patients with PD-L1 positive tumors benefited more from immunotherapy combination as compared to those affected by PD-L1 negative tumors in terms of DFS.

## 5. Current Therapeutic Guidelines in NPC and Future Perspectives

The correlation between EBV infection and NPC development is well established; however, the molecular mechanisms underlying this type of correlation still need to be better defined. Data available from the literature, mostly extrapolated from carcinogenicity studies on B-lymphocytes, demonstrated that some oncoproteins are involved. These oncoproteins are LMP-1, LMP-2, and EBNA1. The latter, as foreign antigens, are potentially able to elicit a specific host immune response against infected tumor cells (transformed epithelial cells of the nasopharynx).

### 5.1. Current Guidelines in NPC

**Figure 4:**
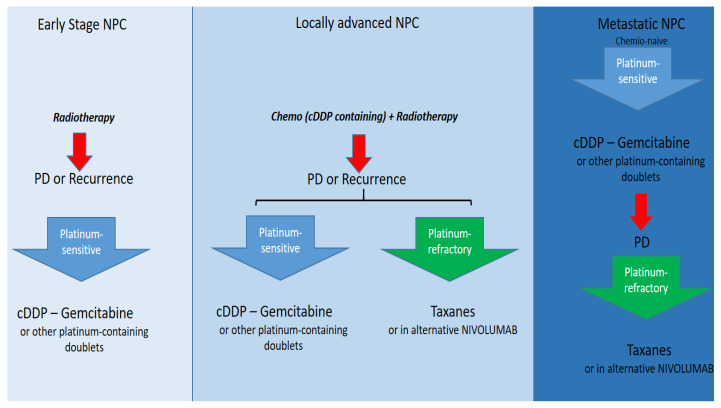
Current therapy options in NPC overall.

Most NPCs are diagnosed as locally advanced disease, and in this case, the standard treatment is concurrent or sequential chemo-radiotherapy [64]. About half of the patients treated with upfront chemo-radiotherapy experience disease recurrence. The prognosis of the latter is poor and the therapeutic possibilities are fewer. In patients not amenable to surgery or re-irradiation at the site of recurrence, systemic therapy is the gold standard. When choosing systemic therapy, it is strictly necessary to distinguish two categories of patients, namely platinum-sensitive and platinum-refractory patients. The first category includes those who have never undergone chemotherapy (chemo-naive) or those who experience a relapse more than 6 months after the last platinum-containing therapy. The second category includes those who have experienced disease progression/relapse within 6 months of their last platinum treatment [65,66]. In patients considered platinum-sensitive, the gold standard is platinum-based polychemotherapy, and in particular “doublets-containing platinum, namely cDDP-5fluorouracil, cDDP-docetaxel, cDDP-gemcitabine, and CBDCA-paclitaxel. The scheme of choice, based on data from randomized trials, is the cDDP-gemcitabine doublet [67]. In platinum-refractory patients, the therapy of choice is monochemotherapy with taxanes (docetaxel or paclitaxel alone), while nivolumab can be used as an alternative [68]. Figure 4 shows a decision algorithm in a patient diagnosed with NPC.

### 5.2. Future Perspectives

Different therapeutic approaches have been aimed at eliciting an adaptive immune response toward EBV oncoproteins utilizing an active or an adoptive immunotherapy. Both therapeutic approaches have not obtained clear and incontrovertible results, so far. As TIME in NPC is highly immunosuppressed and expressed checkpoint molecules, the use of checkpoint inhibitors has been evaluated in several clinical studies (as reported in Table 1). The results obtained were particularly encouraging, so much so that a PD-1 inhibitor (nivolumab) is already used in clinical practice while two other drugs with the same mechanism of action (camrelizumab and toripalimab) have given encouraging results in phase III studies. These drugs will probably soon obtain an approval from the regulatory authorities. In the near future, the combination of checkpoint inhibitors and active and/or adoptive immunotherapy should be implemented.

## Figures and Tables

**Table 1:** Clinical trials with check-point inhibitors enrolling patients with recurrent/metastatic NPC.

Trial/Year	Phase	Setting	Drugs	ORR	PFS	OS
KEYNOTE-028 2017 [68]	I/II	R/M P-ref	Pembrolizumab	26%	6 months	NA
CheckMate 358 2017 [69]	I/II	R/M P-ref	Nivolumab	16%	NA	NA
NCT03097939 2020 [70]	II	R/M P-ref	Nivolumab + Ipilimumab	30%	5 months	NA
CAPTAIN 2020 [71]	II	R/M P-ref	Camrelizumab	28%	4 months	17 months
NCI-9742 2018 [72]	II	R/M P-ref	Nivolumab	21%	3 months	17 months
POLARIS-02 2020 [73]	II	R/M P-ref	Toripalimab	21%	2 months	17 months
NCT026059672 2020 [74]	II	R/M P-ref	Spartalizumab vs. Standard therapy	17% 35%	2 months 7 months	25 months 16 months
KEYNOTE-122 2022 [75]	III	R/M P-ref	Pembrolizumab vs. standard therapy	21% 23%	4 months 6 months	18 months 18 months
JUPITER-02 2022 [63]	III	R/M P-sen	Toriplaimab + GC Placebo + GC	79% 67%	21 months 8 months	NR NR
CAPTAIN-1s 2022 [60]	III	R/M P-sen	Camrelizumab + GC Placebo + GC	87% 81%	11 months 7 months	NR NR
RATIONALE 2022 [76]	III	R/M P-sen	Tislelizumab + GC Placebo + GC	70% 55%	9 months 7 months	NR NR

## Data Availability

Data are reported in the manuscript and at link https://zenodo.org/record/7701183#.ZAWx7nbMK3A.
